# Stabilization of EREG via STT3B-mediated N-glycosylation is critical for PDL1 upregulation and immune evasion in head and neck squamous cell carcinoma

**DOI:** 10.1038/s41368-024-00311-1

**Published:** 2024-07-01

**Authors:** Shengming Xu, Haifeng Wang, Yu Zhu, Yong Han, Liu Liu, Xiangkai Zhang, Jingzhou Hu, Wuchang Zhang, Shengzhong Duan, Jiong Deng, Zhiyuan Zhang, Shuli Liu

**Affiliations:** 1 Department of Oral and Maxillofacial-Head and Neck Oncology, Shanghai Ninth People’s Hospital, Shanghai Jiao Tong University School of Medicine, Shanghai, China; 2 National Clinical Research Center for Oral Diseases, Shanghai Key Laboratory of Stomatology & Shanghai Research Institute of Stomatology, Shanghai, China; 3 Laboratory of Oral Microbiota and Systemic Diseases, College of Stomatology, Shanghai Ninth People’s Hospital, Shanghai Jiao Tong University School of Medicine, Shanghai, China; 4 Research Unit of Oral and Maxillofacial Regenerative Medicine, Chinese Academy of Medical Sciences, Shanghai, China; 5 Department of Stomatology, Zhuji Affiliated Hospital of Wenzhou Medical University, Zhuji, China; 6 Department of Implant Dentistry, Shanghai Ninth People’s Hospital, Shanghai Jiao Tong University School of Medicine, Shanghai, China; 7 Medical Research Center, Binzhou Medical University Hospital, Binzhou, China

**Keywords:** Oral cancer, Cancer microenvironment, Cancer immunotherapy

## Abstract

Dysregulated Epiregulin (EREG) can activate epidermal growth factor receptor (EGFR) and promote tumor progression in head and neck squamous cell carcinoma (HNSCC). However, the mechanisms underlying EREG dysregulation remain largely unknown. Here, we showed that dysregulated EREG was highly associated with enhanced PDL1 in HNSCC tissues. Treatment of HNSCC cells with EREG resulted in upregulated PDL1 via the c-myc pathway. Of note, we found that N-glycosylation of EREG was essential for its stability, membrane location, biological function, and upregulation of its downstream target PDL1 in HNSCC. EREG was glycosylated at N47 via STT3B glycosyltransferases, whereas mutations at N47 site abrogated N-glycosylation and destabilized EREG. Consistently, knockdown of STT3B suppressed glycosylated EREG and inhibited PDL1 in HNSCC cells. Moreover, treatment of HNSCC cells with NGI-1, an inhibitor of STT3B, blocked STT3B-mediated glycosylation of EREG, leading to its degradation and suppression of PDL1. Finally, combination of NGI-1 treatment with anti-PDLl therapy synergistically enhanced the efficacy of immunotherapy of HNSCC in vivo. Taken together, STT3B-mediated N-glycosylation is essential for stabilization of EREG, which mediates PDL1 upregulation and immune evasion in HNSCC.

## Introduction

Head and neck squamous cell carcinoma (HNSCC) represents the seventh leading malignancy worldwide with a low 5-year survival rate and poor prognosis.^1^ Despite a variety of *progresses* in combined modality treatments over the past three decades, the overall survival rate is less than 50% in 5 years for HNSCC patients.^2^ Targeted therapy and immunotherapy *have* emerged as the promising strategies in cancer therapy,^3^ however, immune checkpoint inhibitors such as anti-PD-1/PDL1 therapy *demonstrated* limited responses in HNSCC patients.^4^

PDL1 expression in tumor cells is mainly regulated by two mechanisms.^5^ Firstly, the “extrinsic” mechanisms, in which the cellular immune response is driven by natural killer (NK) cells. *NK* cells could produce pro-inflammatory cytokines, such as γ-interferon from CD8+ tumor-infiltrating T cells, which induce PDL1 expression in tumor cells and mediate immune evasion. *Secondly*, the “intrinsic” mechanisms. Some dysregulated signalings can aberrantly increase PDL1 expression in tumor cells. For example, PTEN deficiency in glioblastoma mediate PDL1 upregulation through the PI3K-AKT pathway.^6^ In lung cancer, EGFR mutations are closely associated with PDL1 dysregulation.^7,8^ However, EGFR mutations are extremely rare in HNSCC, whereas upregulation of wild-type EGFR is prevalent in about 80 to 90% of HNSCCs.^9^ These suggest that the mechanisms underlying dysregulated PDL1 in HNSCC are different from those in lung cancer. Therefore, exploration of the mechanism of PDL1 dysregulation in HNSCC may greatly improve the efficacy of immunotherapy.

Epiregulin (encoded by the EREG gene) is a 169-amino acid protein that belongs to the epidermal growth factor (EGF) family. Epiregulin binds to EGFR (ErbB1) and ErbB4 (HER4) and stimulate ErbB2 (HER2/Neu) and ErbB3 (HER3) signaling through ligand-induced heterodimerization.^10^ Many studies have shown that EREG expression correlates with cancer metastasis and may serve as a prognostic marker for cancers such as breast, colorectal, bladder and oral cancers.^11^ Previously, we have shown that EREG overexpression promotes oncogenesis in HNSCC via inducing c-Myc.^12^ Importantly, EREG is co-expressed with PDL1 with significant functionality.^13^ Moreover, the upregulated EREG gene is strongly associated with the dysregulated immune reesponse and inflammatory responses.^14^ Alternatively, dysregulated EREG is implicated in the immune evasion in HNSCC, which may serve as a *potential* target in cancer immunotherapy. However, the role and molecular mechanisms underlying EREG dysregulation remain elusive.

Glycosylation is a common posttranslational modification, and is involved in regulation of many cellular activities, including proliferation, migration, adhesion and apoptosis.^15^ Glycosylation is completed through collaboration between cellular glycosyltransferases and glycosidase, which add or trim glycans on asparagine (N-linked glycan) or serine/threonine (O-linked glycan) residues in polypeptides in the endoplasmic reticulum and the Golgi apparatus.^16,17^ The biochemical functions of N-glycan attachment to a glycoprotein, which occurs in the Asn-X-Ser/Thr consensus peptide sequence. N-glycosylation influences protein solubility, stabilization, and receptor-ligand interaction.^18^ Further process of N-glycosylation takes place in the Golgi apparatus via a sequential glycosidase- and glycotransferase-mediated glycoprotein biosynthesis.^19^ The STT3 protein is the central enzyme of oligosaccharyl transferase (OST) complex, and two isoforms exist in mammalian cells, namely, STT3A and STT3B.^20^ The STT3 isoforms initiate N-glycosylation by catalyzing the transfer of glycan from dolichol to the asparagine residues of substrates.^20,21^ Previous study have revealed a model by which the two STT3 isoforms act sequentially on polypeptides to maximize the efficiency of N-glycosylation.^22^ Although STT3 is critical for EMT-mediated PDL1 upregulation in breast cancer,^23^ its role in tumorigenesis is not well understood, especially for STT3B.

In this study, we investigated the mechanism of EREG stability mediation, which is mediated by STT3B-mediated (N)-linked glycosylation. Thus, with combination of targeting the oligosaccharyl transferase (OST) complex, which is essential for EREG stabilization, the efficacy of anti-PDL1 therapy can be greatly improved.

## Results

### Dysregulated EREG upregulates PDL1 in human HNSCC cells

**Fig. 1:**
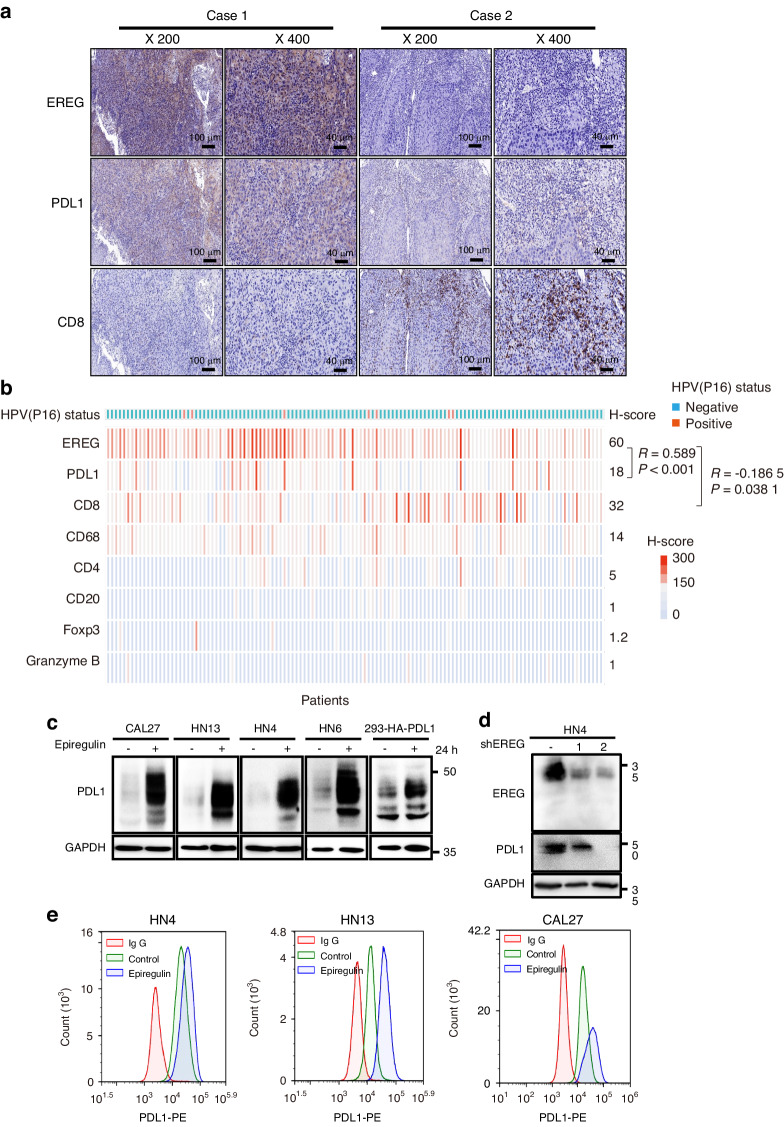
Dysregulated EREG up-regulates PDL1 expression in human HNSCC cells. **a** Representative images of EREG staining in two patients with PDL1 and CD8 expression. Case 1 showed high expression of EREG with high expression of PDL1 and low expression of CD8. Case 2 showed low expression of EREG with low expression of PDL1 and high expression of CD8. **b** Hotmap of H-score of EREG and other immune-related markers in a cohort of 124 HNSCC tissues. Statistical analysis of IHC staining indicated that EREG expression is positively correlated with PDL1 (*R* = 0.589 0, *P* < 0.001) and negatively correlated with EREG (*R* = −0.186 5, *P* < 0.001) expression in HNSCC tissues. **c** Western blot analysis of PDL1 from four different HNSCC cell lines treated with 50 ng/mL epiregulin for 24 has indicated and HA-PDL1 from HEK293 cells treated with 50 ng/mL epiregulin for 24 h. **d** Western blot analysis of PDL1 expression in shControl and two independent shEREG stable clones of HN4 cells. **e** Cell surface analysis of PDL1 protein in three different HNSCC cell lines using flow cytometry

To determine the relationship of EREG in tumor evasion in HNSCC, we analyzed the “Lymphocyte”, “Immunomodulator”, and “Chemokine” tab respectively from the Tumor and Immune System Interaction Database (TISIDB) database to evaluate whether EREG might regulate the immune features across multiple cancers. The landscape of the relationship between EREG expression and 28 types of tumor-infiltrating lymphocytes in different types of cancer showed that EREG expression was negatively correlated with the infiltrated Act CD8 (*r* = −0.115, *P* = 0.008 68) and Act CD4 (*r* = −0.203, *P* < 0.01) cells in HNSCC (Supplementary Fig. 1a, b), and positively correlated with infiltrated neutrophils cells (*r* = 0.567, *P* < 0.01) and monocytes (*r* = 0.186, *P* < 0.01) in HNSCC (Supplementary Fig. 1a, b). Immunomodulators can be further classified into immunoinhibitors, immunostimulators, and major histocompatibility complex (MHC) molecules. EREG expression was correlated with immunoinhibitors (Supplementary Fig. 1c), of which the greatest correlations included CD274 (*r* = 0.115, *P* = 0.008 55), LGALS9 (Spearman: *r* = −0.429, *P* < 2.2e-16), TGFB1 (*r* = 0.363, *P* = 3.26e-18), and VTCN1 (*r* = −0.313, *P* = 3.39e-13) in HNSCC (Supplementary Fig. 1d). In addition, analysis of the relationship between EREG expression and immune inhibitors and MHC molecules revealed that the expression of EREG positively correlated with NT5E (*r* = 0.519, *P* < 2.2e-16), PVR (*r* = 0.382, *P* < 2.2e-16) and RAET1E (*r* = 0.503, *P* < 2.2e-16) (Supplementary Fig. 1e, f, g). For chemokines, the expression of EREG was negatively correlated with CX3CL1 (*r* = −0.452, *P* < 2.2e-16), CXCR4 (*r* = −0.275, *P* = 1.86e-10), and CCR10 (*r* = −0.246, *P* = 1.41e-08), and positively correlated with CXCL1 (*r* = 0.365, *P* = 1.03e-18), CXCL8 (*r* = 0.423, *P* < 2.2e-16), CXCR1 (*r* = 0.466, *P* < 2.2e-16), and CXCR2 (*r* = 0.378, *P* < 2.2e-16) (Supplementary Fig. 1h–k). Taken together, these data suggested that dysregulated EREG is involved in regulating the immunomodulators. To validate the relationship between EREG, PDL1, and CD8, immunohistochemistry (IHC) array was performed on a microtissue array of 124 head and neck squamous cell carcinomas. A positive correlation was found between EREG and PDL1 expression (*R* = 0.589, *P* < 0.001, Fig. 1a, b)*,* while a negative relationship was also observed between EREG and CD8 expression (*R* = −0.186 5, *P* = 0.038 1, Fig. 1a, b). We further investigated the differences in expression and significance of EREG in comparison with HPV (−) and HPV(+) HNSCC by using TIMER which is based on TCGA database. The expression level of EREG in HPV (−) HNSCC is higher than that of HPV (+) HNSCC (Supplementary Fig. 1l). We also tested HPV status of all 124 sample and found only 5.6% samples were HPV positive (7/124). The H-score of EREG in HPV (+) samples was not significantly different compared to HPV(−) samples, perhaps due to limited number of HPV(+)HNSCC samples (Supplementary Fig. 1m). Furthermore, we investigated the activation state of CD8(+) T cell by performing Granzyme B immunohistochemistry staining on 124 HNSCC samples, however, the expression of Granzyme B was relatively low (Fig. 1b, Supplementary Fig. 1n). We also investigated the role of regulatory T cells by performing Foxp3 immunohistochemistry staining, and it also exhibited low expression (Fig. 1b, Supplementary Fig. 1n). To investigate functionality of other immune cells such as B cells and macrophages, we performed CD4, CD20, CD68 staining, and no association was identified between EREG expression and these immune cells (Fig. 1b, Supplementary Fig. 1n). All these results showed that EREG was correlated with PDL1 whereas was inversely correlated with CD8 T-cell infiltration.

Next, we examined EREG and PDL1 in a panel of 10 HNSCC cell lines. Of note, EREG was strongly correlated with PDL1 in these cell lines (Supplementary Fig. 1o–q). These findings suggest that dysregulated EREG is involved in upregulation of PDL1 in HNSCC. Next, we examined the role of EREG in regulation of PDL1. Treatment with rhEREG (recombinant homo epiregulin) or autocrined EREG in conditioned medium (CM) both significantly increased PDL1 in different HNSCC cell lines (Fig. 1c, Supplementary Fig. 1s). Consistently, EREG treatment also enhanced cell-surface PDL1 in HN4, HN13, and CAL27 cells (Fig. 1e). In addition, immunofluorescence (IF) staining showed that the intensity of PDL1 was significantly increased in HN13 cells treated with EREG compared to those in control cells (Supplementary Fig. 1r). Importantly, knockdown of EREG via lentiviral short-hairpin RNA (shRNA) or specific siRNAs significantly downregulated PDL1 protein levels in both HN4 and HN30 cells compared to that in control cells (Fig. 1d, Supplementary Fig. 1t). Moreover, we examined the roles of several growth factors and inflammatory cytokines in PDL1 expression HNSCC cells, such as epidermal growth factor (EGF), LPS, IL-6, IL-1β, and TNF-α, commonly in tumor microenvironment. Of note, only EGFR ligand EREG and EGF are strong inducers for PDL1 expression in HN13 cells (Supplementary Fig. 1u). Taken together, these observations suggest that EREG plays a critical role in PDL1 upregulation in HNSCC cells.

### EREG upregulates PDL1 via the EGFR-c-Myc pathway

**Fig. 2:**
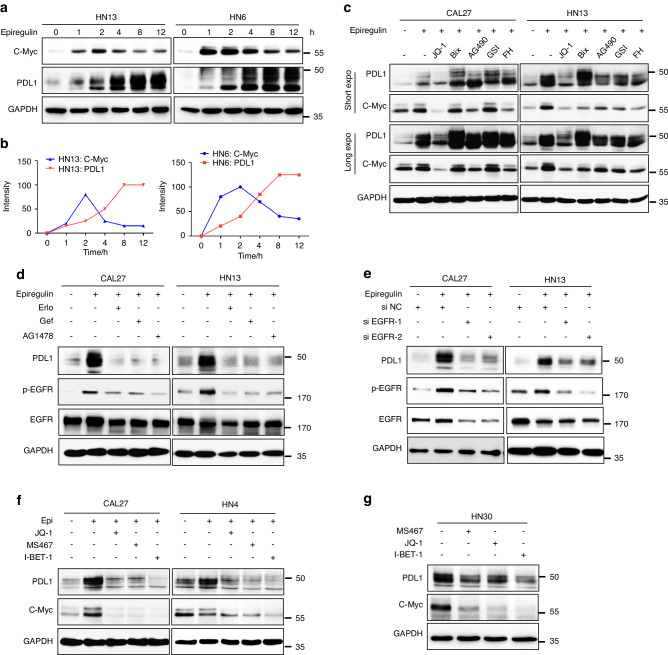
EREG upregulates PDL1 through c-Myc. **a** Western blot analysis of PDL1 and c-Myc expression in HN6 and HN13 cells after treatment with EREG for different time intervals. **b** Plot of densitometry results for the levels of PDL1 and c-Myc. **c** CAL27 and HN13 cells were pretreated with various inhibitors for 1 h followed by stimulation with epiregulin for 24 h. The levels of PDL1 and c-Myc were examined by Western blot analysis. **d** Western blot analysis of PDL1, p-EGFR, EGFR, and GAPDH expression in CAL27 and HN13 cells pretreated with various EGFR inhibitors for 1 h followed by stimulation with epiregulin for 24 h. **e** Twenty-four hours after transfection with si-EGFR or si-NC short interfering RNAs (siRNAs), cells were further treated with EREG for 24 h, and the expression of PDL1, p-EGFR, EGFR, and GAPDH was detected by Western blot. **f** CAL27 and HN4 cells were pretreated with various BET BD inhibitors for 1 h followed by stimulation with epiregulin for 24 h. The levels of PDL1 and c-Myc were examined by Western blot analysis. **g** HN30 cells were treated with various BET BD inhibitors for 24 h, and the levels of PDL1 and c-Myc were examined by Western blot analysis

Dysregulated EREG has been showed to promote HNSCC oncogenesis via the c-Myc pathway. Since Myc binds to the promoter of PDL1 for increased transcription,^24^ we hypothesized that EREG upregulates PDL1 through c-Myc. We examined the roles of EREG on c-Myc and PDL1 in HNSCC cells. The results showed that rhEREG (epiregulin) treatment significantly increased both c-Myc and PDL1 in two HNSCC cell lines (Fig. 2a). Kinetically, the induction of c-Myc occurred earlier than the induction of PDL1 (Fig. 2b), suggesting that PDL1 induction is mediated by c-Myc expression. To determine the role c-Myc in EREG-induced PDL1, we treated cells with EREG or EREG plus c-Myc inhibitor, JQ-1. Importantly, JQ-1 significantly suppressed EREG-induced PDL1 whereas treatment with other inhibitors, including Bix, AG490, GSI, and FH, did not (Fig. 2c). These suggest that EREG induces PDL1 upregulation in a c-Myc-dependent manner in HNSCC.

To determine if the EGFR kinase activity is essential for EREG-induced PDL1, we examined the roles of EREG in absence or presence of EGFR inhibitors, erlotinib, gefitinib, or AG1478 in HNSCC cells. The results showed that EREG-induced PDL1 was significantly suppressed in presence of EGFR inhibitors, suggesting that activated EGFR is essential for PDL1 induction in HNSCC cells (Fig. 2d). Consistently, knockdown of EGFR with siRNA-EGFR significantly suppressed EREG-induced PDL1 in HNSCC cells (Fig. 2e, Supplementary Fig. 2a, b). It has been shown that suppression of bromodomain-containing protein 4 (BRD4) via pharmacological BET-specific bromodomain (BD) inhibitors can effectively block Myc expression in multiple cancers.^25^ To determine the role of BRD4, we examined the effect of BET inhibition on EREG-mediated PDL1 induction. Pretreatment with three different BET inhibitors, JQ-1, MS467 and I-BET-1, greatly suppressed EREG-induced PDL1 in both CAL27 and HN4 cells (Fig. 2f). Moreover, treatment with BET inhibitors also blocked PDL1 and c-Myc expression in HN30, in which PDL1 is persistently upregulated (Fig. 2g). These results suggest that the EGFR-c-Myc pathway is essential for EREG-mediated PDL1 upregulation.

In addition, we searched for genomic alterations in the EREG-EGFR-PDL1 pathway from the TCGA data via the cBio Cancer Genomics Portal as previously described.^11^ OncoPrint analysis showed that alterations in genes from the EREG-EGFR-CD274 pathway were mutually exclusive (Supplementary Fig. 2c), suggesting that overexpression of these genes has a similar functional role. Moreover, patients with EREG/EGFR/PDL1 alterations showed a significantly worse overall survival rate than those without (Supplementary Fig. 2d, e). Taken together, these results supported that EREG upregulates PDL1 via the EGFR-c-myc pathway in HNSCC.

### EREG is N-glycosylated in HNSCC cells

**Fig. 3:**
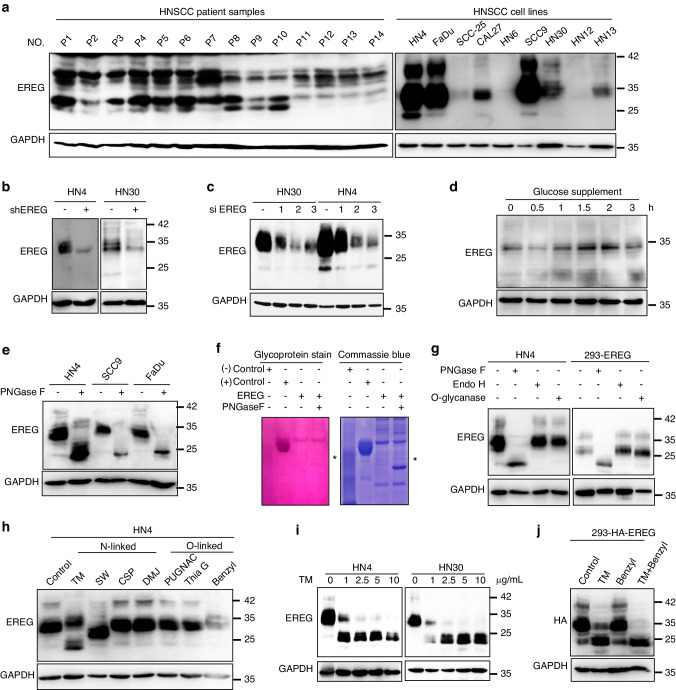
EREG is glycosylated in HNSCC cancer cells. **a** Expression of EREG protein in primary HNSCC patient samples and cell lines. Western blot analysis of EREG in 14 representative HNSCC patient samples and 9 HNSCC cell lines. **b** Western blot analysis of EREG expression in shControl and shEREG stable clones of HN4 and HN30 cells. **c** Western blot analysis of EREG expression in siNC and three individual small interfering RNAs (siRNAs) targeting EREG in both HN4 and HN30 cells. **d** Western blot analysis of EREG expression in HN4 cells with glucose supplementation (5.55 mmol/L) at the indicated times. HN4 cells were cultured in serum-free medium with a low concentration of glucose (5.55 mmol/L) for 24 h before glucose supplemention. **e** Glycosylation pattern of EREG protein in HN4, SCC9 and FaDu cells. Cell lysates were treated with PNGase F and analyzed by Western blot analysis. Black circles indicate glycosylated EREG, and arrowheads indicate non-glycosylated EREG. **f** Glycoprotein staining and Coomassie blue staining of PNGase F-treated purified EREG. Horseradish peroxidase (HRP) and soybean trypsin inhibitor (STI) served as positive and negative controls, respectively. **g** Cell lysates from the indicated cell lines were treated with PNGase F, Endo H, and O-glycanase for 1 h at 37 °C in vitro. **h** Immunoblot of EREG in HN4 cells treated with inhibitors blocking N-linked or O linked glycosylation as indicated. **i** Immunoblot of EREG in HN4 and HN30 cells treated with the N-linked glycosylation inhibitors TM as indicated. **j** Immunoblot of EREG in HEK293-EREG cells treated with the N-linked glycosylation inhibitor TM or the O-linked glycosylation inhibitor benzyl as indicated

The molecular weight of EREG is predicted to be ~25 kD. Interestingly, the majority of EREG was >30 kD in both human HNSCC tissues and HNSCC cell lines. The molecular pattern suggests that EREG is glycosylated (Fig. 3a). The immunoblot analysis showed that the predicted-glycosylated, and -unglycosylated bands are indeed EREG, since knocking down of EREG by lentiviral short-hairpin RNA (shRNA) or by 3 specific siRNAs reduced both the 30- and 25-kD bands of EREG (Fig. 3b, c). Moreover, when cultured in a low concentration of glucose (5.55 mmol/L) for 24 h, glucose supplementation rapidly increased the 30-kD form of EREG, suggesting that glucose uptake provided a substrate for glycosylation and enhanced the glycosylation levels of EREG (Fig. 3d). Importantly, when HNSCC cell lysates (HN4, SCC9, and FaDu) treated with recombinant peptide-N-glycosidase F (PNGase F) glycosidase, which removes the entire N-glycan structure in vitro, glycosylated EREG (30 kD) was degraded into unglycosylated form (20 kD) (Fig. 3e). In addition, EREG was purified from 293 T cells, treated with or without PNGase F and then run SDS–PAGE for either glycoprotein staining or commassie blue staining. The results showed that PNGase F treatment reduced the molecular weight of EREG (Fig. 3f), confirming that the high MW (30 kD) of EREG is the glycosylated form, and the low MW (25 kD) of EREG was the unglycosylated form, resulted from PNGase F treatment. Of note, treatment with glycosidase F effectively removed glycosylation, whereas treatment with endoglycosidase H (Endo H), or recombinant O-glycosidase did not in HN4 cells and 293-EREG cells (Fig. 3g). These results suggest that glycosylation on EREG is predominantly N-linked glycan structures. Moreover, treatment with tunicamycin (TM) and swainsonine (SW), the inhibitors of N-linked glycosylation, also reduced MW of EREG (Fig. 3h, i, Supplementary Fig. 3a), whereas treatment with inhibitors of O-linked glycosylation, e.g., benzyl-GalNAc, did not (Fig. 3h, j; Supplementary Fig. 3b). These results indicated that EREG in HNSCC cells is primarily N-linked glycosylated.

### N-glycosylation mediates stabilization of EREG in HNSCC cells

**Fig. 4:**
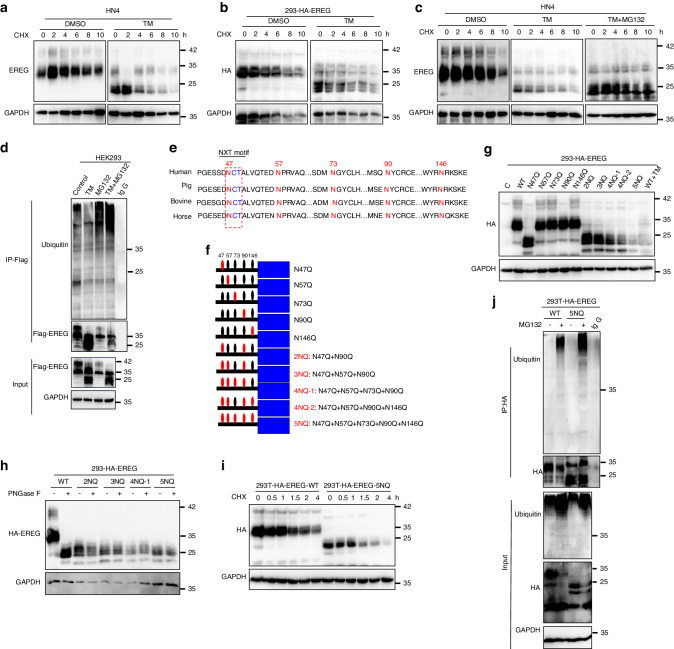
N-glycosylation of EREG is critical for stabilization of EREG in HNSCC cells. Deglycosylation of EREG enhanced the turnover of EREG. HN4 (**a**) and 293-EREG (**b**) cells were treated with 10 μg/mL tunicamycin (N-glycosylation inhibitor) for 24 h followed by pulse-chase with 100 μg/mL cycloheximide. Protein levels at the indicated time points were evaluated by immunoblot analysis. The intensity of the glycosylated form versus the nonglycosylated form of EREG was quantified using ImageJ software. **c** Immunoblot of EREG in HN4 cells treated with CHX for the indicated time in the presence or absence of TM and MG132. **d** HEK293 cells were transfected with Flag-EREG in the presence or absence of MG132 and/or TM. Flag-EREG was then immunoprecipitated followed by immunoblotting using anti-ubiquitin antibody. **e** Schematic diagram of EREG amino acid sequence alignment among different species. The NXT motif is shown in blue. **f** Predicted N-glycosylation sites of human EREG by NetNGlyc1.0 Server. **g** Western blot analysis of the protein expression pattern of EREG WT and its NQ mutants. The nonglycosylated form in Lane 13 indicates EREG-WT with overnight treatment with TM. **h** Western blot analysis of the protein expression pattern of EREG WT and its NQ mutants. Cell lysates were treated with PNGase F and analyzed by Western blot. **i** The indicated cell lines were treated with CHX at the indicated intervals. The intensity of EREG protein was quantified using ImageJ software. **j** Ubiquitination of EREG proteins in EREG-WT- or EREG-5NQ mutant-expressing HEK293 cells. EREG proteins were immunoprecipitated with HA antibody and then immunoblotted with ubiquitin antibody

Since the level of glycosylated EREG were significantly higher than that of its non-glycosylated form in HNSCC cells (Fig. 3a), we asked if glycosylation affects EREG stability by using a pulse-chase assay with cycloheximide, an inhibitor of protein biosynthesis. The results showed that the highly glycosylated form had a half-life >4 h whereas the unglycosylated forms of EREG had a half-life <1 h (Supplementary Fig. 4a, b). Moreover, treatment with proteasome inhibitor MG132 increased ubiquitin conjugates of EREG (Supplementary Fig. 4c), suggesting that EREG turnover is mediated by the ubiquitin–proteasome pathway. In presence of cycloheximide (CHX), non-glycosylated EREG, which was induced by TM, exhibited a faster turnover rate than glycosylated EREG in both HN4 and HEK293T cells (Fig. 4a, b; Supplementary Fig. 4d). These suggest that glycosylation enhances the stability of EREG. Pretreatment with MG132 slowed down degradation rates of non-glycosylated EREG, whereas treatment with chloroquine (CQ) lysosome inhibitor did not, suggesting that both glycosylated and nonglycosylated EREG were degraded via the proteasome-dependent pathway (Fig. 4c, Supplementary Fig. 4e–h). The effects of glycosylation on EREG ubiquitination were confirmed in HEK293T and HN4 cell lines by IP-Flag-EREG for blotting ubiquitin (Fig. 4d; Supplementary Fig. 4i). Taken together, these results suggest that N-glycosylation mediates stabilization of EREG, preventing from the ubiquitination-dependent degradation.

To define the glycosylation sites on EREG, we searched for evolutionarily conserved NXT motifs in the EREG amino acid sequences from different species, and found one NXT motif in five potential N-glycosylation sites (Fig. 4e; Supplementary Fig. 4j). The potential thresholds of all of these sites were predicted over 0.5 (Supplementary Fig. 4k). To determine the biochemical features of the sites, we generated a series of EREG mutants (N to Q), expressed them in HEK293T cells, and performed Western blot analysis. Compared to the wild-type (WT) EREG, N47Q had significantly reduced EREG glycosylation. The majority of the N47Q mutant was unglycosylated and only left a small portion above 25 kD (Fig. 4f, g, lane 3). On contrast, no significant difference in glycosylation was observed in N57Q, N73Q, N90Q, and N146Q mutants (Fig. 4g). Importantly, EREG glycosylation was completely ablated in the EREG mutant bearing 2NQ, 3NQ, 4NQ, and 5NQ (Fig. 4g, h). Taken together, these results demonstrate that EREG is N-glycosylated at N47. Further experiments showed that the degradation rate of non-glycosylated EREG in the EREG-5NQ mutant was faster than that of glycosylated EREG in EREG-WT (Fig. 4i; Supplementary Fig. 4l). In presence of MG132, EREG-5NQ exhibited higher levels of ubiquitination (Fig. 4j). These results suggested that unglycosylated EREG is susceptible to the ubiquitin-mediated degradation.

### N-glycosylation of EREG is critical for maintaining membrane location and autocrine activity

**Fig. 5:**
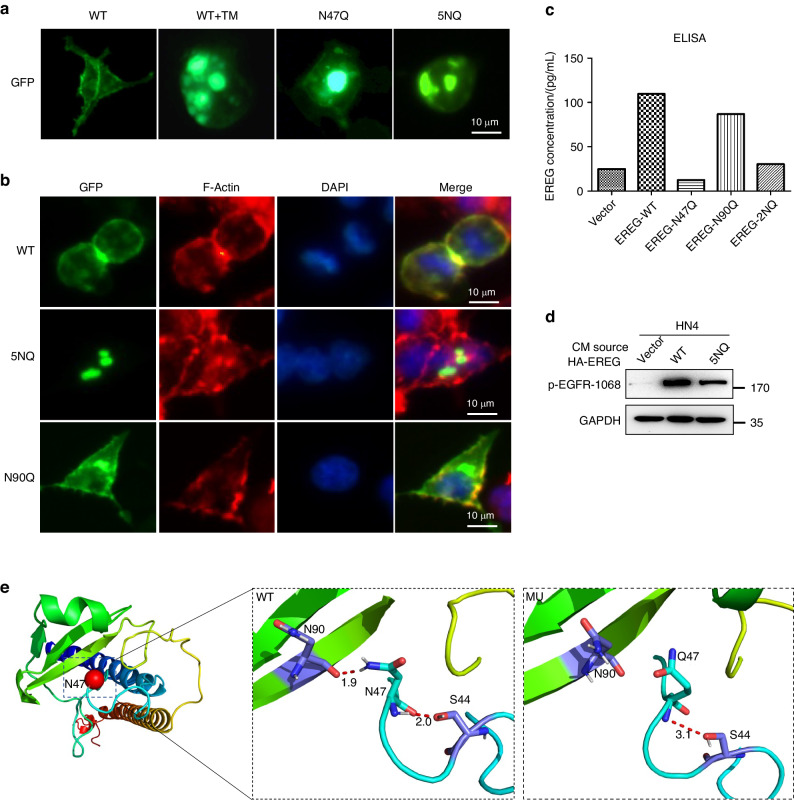
Glycosylation of EREG is crucial for maintaining its membrane subcellular location and autocrine activity. **a** GFP-EREG localization in HEK293 cells expressing WT, WT + TM, N47Q, or 5NQ mutant EREG by IF staining. **b** Colocalization of EREG and F-actin in HEK293 cells expressing WT, 5NQ, or N90Q mutant EREG by IF staining. **c** ELISA of EREG levels in conditioned medium from HEK293 cells expressing WT, N47Q, N90Q, or 2NQ mutant EREG. **d** HN4 cells were treated for 5 min with CM from HEK293 cells expressing vector, WT, or 5NQ mutant and analyzed by WB with the indicated antibodies. **e** Protein structure of mutant N47Q. The N and O atoms of the polar uncharged asparagine at position 47 of the wild-type protein form hydrogen bonds with the polar uncharged serine O atom at position 44 and the polar uncharged asparagine O atom at position 90. The hydrogen bond distances are 2.0 and 1.9 Å. The N47Q mutation results in the substitution of the polar uncharged asparagine amino acid at position 47 by the polar uncharged glutamine. After the mutation to glutamine, it forms a hydrogen bond with the non-polar serine O atom at position 44. The hydrogen bond distance is 3.1 Å, and it cannot interact with the N90 amino acid in polarity. The changes in the interaction of these amino acids may lead to changes in the protein structure and functional components after the mutation

Next, we asked if glycosylation alters the distribution of EREG to the membrane. IF analysis showed that GFP-EREG-WT and -N90Q were mainly localized on cell membrane (Fig. 5a, b). In contrast, WT treated with TM, which blocks N-linked glycosylation, or N47Q or 5NQ mutants, EREG were mainly located in cytoplasm (Fig. 5a, b). To determine the effects of glycosylation on autocrine excretion of EREG, we examined the released EREG via autocrine in CM from EREG-WT, EREG-N47Q, N90Q, and 2NQ cells. As expected, glycosylated EREG (WT) and N90Q exhibited a higher EREG concentration than unglycosylated EREG (N47Q and 2NQ) (Fig. 5c). Western blot showed that HN4 cells treated with CM from WT-EREG exhibited high level of EGFR activation compared to that from 5NQ mutant (Fig. 5d). These findings suggested that N-glycosylation of EREG is critical for maintaining its membrane expression and biological activity via autocrine. A predicated model structure of EREG-WT and EREG-N47Q also showed that the glycosylated EREG at N47 is critical for the EREG protein structure and function (Fig. 5e, Supplementary Fig. 5a, b).

### N-glycosylation of EREG is catalyzed by STT3B

**Fig. 6:**
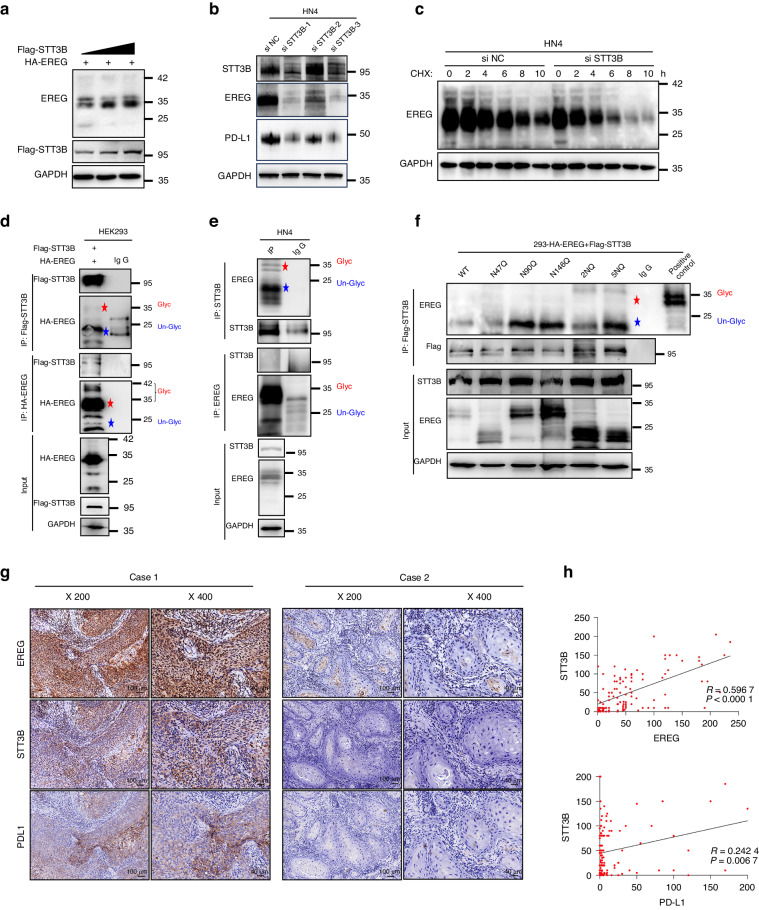
The glycosylation of EREG is induced by STT3B. **a** HA-EREG was co-expressed with increasing amounts of Flag-STT3B in HEK293 cells. Lysates were subjected to Western blot analysis. **b** Western blot analysis of STT3B, EREG and PDL1 expression in HN4 cells after transfection with siSTT3B or siNC siRNAs. **c** HN4 cells were transfected with control or STT3B siRNA. After cells being treated with CHX, the expression of endogenous EREG was analyzed by Western blotting. **d** HEK293 cells were transiently co-transfected with Flag-STT3B and HA-EREG. Cell extracts were immunoprecipitated separately with anti-Flag or anti-HA antibodies, and the associated EREG and STT3B proteins were examined by Western blotting. Red pentagram symbol indicates glycosylated EREG, blue pentagram symbol indicates un-glycosylated EREG. **e** Endogenous EREG and STT3B were immunoprecipitated from HN4 cells, and bound endogenous STT3B and EREG were examined by Western blotting. Red pentagram symbol indicates glycosylated EREG, blue pentagram symbol indicates un-glycosylated EREG. **f** HEK293 cells were transiently cotransfected with Flag-STT3B and different HA-EREG mutants (WT, N47Q, N90Q, N146Q, 2NQ, and 5NQ). Cell extracts were immunoprecipitated separately with anti-Flag antibodies, and the associated EREG proteins were examined by Western blotting. Red pentagram symbol indicates glycosylated EREG, blue pentagram symbol indicates un-glycosylated EREG. **g** Representative paired immunohistochemistry staining of EREG, STT3B and PDL1. **h** Statistical analysis of immunohistochemistry staining of the tissue array showed that both EREG and PDL1 expression are positively correlated with STT3B expression in HNSCC (*P* < 0.001)

To determine if glycosyltransferases is responsible for N-glycosylation of EREG, we searched for the interacted proteins. EREG from Flag-EREG HEK293 cells was purified by affinity capture purification (Supplementary Fig. 6a) and then subjected to Mass spectrometric analysis for the EREG interactome. Within this interactome, several proteins such as STT3B, RPN1, and GNPAT as EREG candidate glycosyltransferases were identified. By analysis of the correlation between EREG and these glycosyltransferase genes in HNSCC patients using data from TCGA, we found that only STT3B was closely and positively correlated with EREG in HNSCC tissues (Supplementary Fig. 6b), and Western blot suggested that EREG and STT3B proteins expression showed positive correlation in HNSCC cell lines (Supplementary Fig. 6c). Then, we investigated if EREG glycosylation is catalyzed by STT3B in HNSCC. Glycosylated EREG was enhanced by increasing STT3B expression in a dose-dependent manner (Fig. 6a). Knockdown of STT3B resulted in reduced EREG and PDL1 in HN4 cells (Fig. 6b). To determine the effect of STT3B-mediated glycosylation on the stability of EREG, a cycloheximide pulse-chase assay was performed. STT3B knockdown increased EREG turnover in HN4 cells (Fig. 6c; Supplementary Fig. 6d). These results suggest that STT3B is required for glycosylation and stabilization of EREG in HNSCC.

To determine if EREG interacts with STT3B, we performed IP-Westernblot following co-expression of HA-EREG and Flag-STT3B in HEK293 cells. Immunoprecipitating STT3B, associated EREG was detected, and vice versa (Fig. 6d). Similarly, immunoprecipitating endogenous STT3B or EREG, detected EREG or STT3B, respectively in HN4 cells (Fig. 6e). Of note, EREG, both exogenous and endogenous, that interacted with STT3B was mainly in non-glycosylation form (Fig. 6d, e), suggesting that STT3B binds to the non-glycosylated EREG and then promotes its glycosylation. In addition, we also performed IP-Westernblot following co-expression of various mutants of HA-EREG (WT, N47Q, N90Q, N146Q, 2NQ and 5NQ) and Flag-STT3B in HEK293 cells. The results showed that all of the EREG mutants, in the nonglycosylated EREG form, interacted with STT3B (Fig. 6f). To determine the clinical relevance of STT3B-mediated EREG glycosylation in vivo, we examined the EREG, STT3B and PDL1 in HNSCC samples by IHC staining. The results showed that there was a strong correlation between the expression levels of STT3B and EREG (*P* < 0.001) and the expression levels of STT3B and PDL1 (*P* < 0.01) (Fig. 6g, h). Taken together, these results suggest that EREG glycosylation is mediated by STT3B, and the expression of EREG and STT3B is strong correlated with dysregulated PDL1 in HNSCC.

### Pharmacological suppression of EREG glycosylation enhances the efficacy of anti-PDLl blockade in vivo

**Fig. 7:**
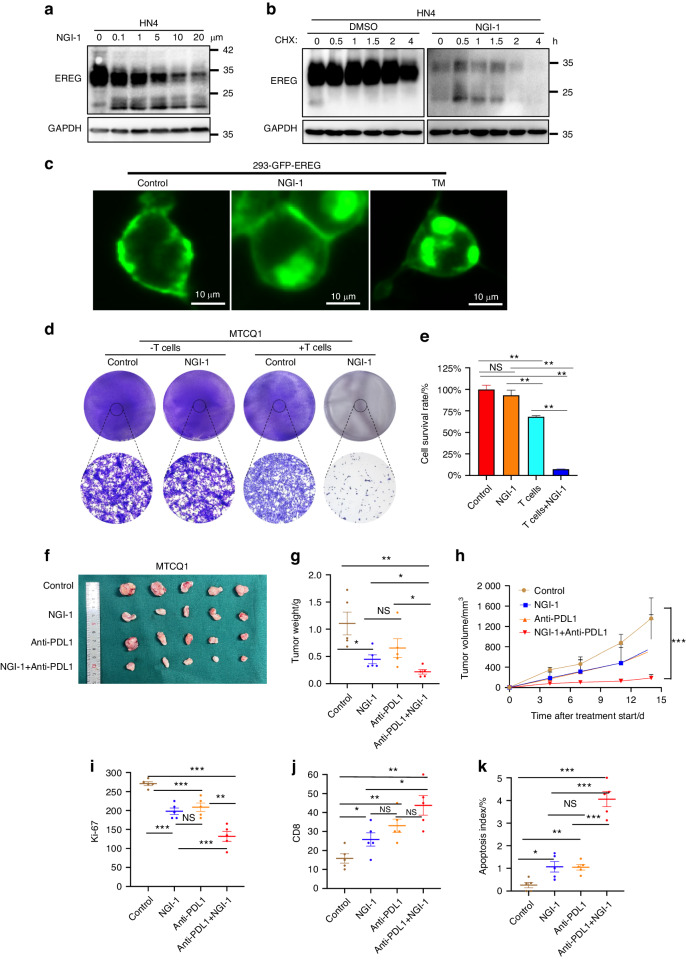
STT3B inhibitor suppresses EREG glycosylation and enhances the efficacy of PD-1 blockade in vivo. **a** HN4 cells were treated with 0-20 μmol/L NGI-1 (OST inhibitor) for 24 h. The expression of EREG was examined by immunoblotting. **b** HN4 cells were treated with or without NGI-1 followed by treatment with CHX, and the expression of endogenous EREG was analyzed by Western blotting. **c** GFP-EREG localization in HEK293 cells treated with NGI-1 or TM by IF staining. **d**, **e** T cell-mediated cytotoxicity was assessed in MTCQ1 cells with or without the NGI-1 treatment (n = 5). ns, not significant; *P < 0.05; **P < 0.01. **f** Mouse MTCQ1 cells were injected into C57 mice. When established tumors were palpable, the mice were treated with vehicle, NGI-1, anti-PDL1 mAb, or NGI-1 + anti-PDL1 mAb (n = 5) via i.p. injection. MTCQ1 tumors in each group were harvested and photographed at the end of the experiment. Photographs of the xenograft tumors are shown. **g** Tumor weights were measured for each treatment group at autopsy. **h** Tumors were measured with calipers, and values were plotted. The vertical bars indicate the mean tumor size (mm^3^) ± SE. **i** IHC scores for Ki-67 expression in tumor sections from each treatment group. (*P < 0.05, **P < 0.01, and ***P < 0.001). **j** IHC scores for CD8 expression in tumor sections from each treatment group. (*P < 0.05, **P < 0.01). **k** Quantitative analysis of TUNEL-positive cells among groups. (n = 5, *P < 0.05)

Glycosylated EREG via STT3B is critical for stabilization of PDL1, suggesting that the enzymes for N-glycosylation is a potential target for cancer therapy. Oligosaccharyl transferase (OST) is for reversibly regulating N-linked glycosylation in mammalian cells. OST is a hetero-oligomeric enzyme that exists in multiple isoforms and transfers oligosaccharides to recipient proteins. NGI-1, a reversible catalytic subunit inhibitor of the OST, has higher specificity for STT3B than for STT3A.^26^ Since EREG acts as a key driver of oncogenesis and survival signaling in HNSCC, we asked the biochemical consequences of OST inhibition on EREG function. NGI-1 (Supplementary Fig. 7a) blocked EREG N-linked glycosylation in both HN4 and 293-EREG cells as high MW EREG was decreased on immunoblot (Fig. 7a; Supplementary Fig. 7b). In comparison, the mRNA level of EREG did not significantly change following NGI-1 treatment in HN4 cells (Supplementary Fig. 7b). We also compared the effect of NGI-1 on the stability of EREG using a cycloheximide pulse-chase assay. The results showed that pretreatment with NGI-1 increased EREG protein turnover (Fig. 7b; Supplementary Fig. 7d). Moreover, blocking N-linked glycosylation by NGI-1 treatment significantly reduced the membrane EREG and increased the cytosolic EREG (Fig. 7c). These suggest that pharmacologic inhibition of glucosyltransferase STT3B results in significantly increased degradation of EREG.

To determine whether NGI-1-mediated EREG destabilization affects cancer cell immunosuppression, we compared the immunosuppression activity of control and NGI-1 treatment both in vitro and in vivo. Consistently, the MTCQ1 cells treated with NGI-1 were less resistant to mouse T-cell-mediated cytolysis than were the cells with control treatment (Fig. 7d, e). Since EREG-mediated stabilization of PDL1 is critical for immune evasion in HNSCC, next we asked whether targeting EREG glycosylation via NGI-1 would enhance the antitumor efficacy of PDL1/PD-1 blockade therapy in vivo. Four groups, untreated control, anti-PDL1 alone, NGI-1 alone, and combination of NGI-1 and anti-PDL1 treatments were used for evaluation of therapeutic efficacy in the MTCQ1 syngeneic mouse model. While PDL1 blockade or NGI-1 as a single-agent approach showed some difference in tumor progression versus control treatment, combined treatment showed a significantly *superior* therapeutic benefit than either monotherapy (Fig. 7f–h). Importantly, there were significantly fewer Ki-67-positive tumor cells with the combined treatment than with either treatment alone (Fig. 7i and Supplementary Fig. 7e). By IHC staining, we found that the NGI-1 or PDL1 monotherapy and combined treatment groups showed markedly enhanced infiltration of CD8+ T cells into tumors compared to control group (Fig. 7j and Supplementary Fig. 7e). The TUNEL assay showed that apoptotic cells were significantly increased in tumor sections in the combination group compared to other groups, and those in monotherapy were increased compared to control group (Fig. 7k and Supplementary Fig. 7f). In addition, there is no toxicity observed in lung, liver, or heart in therapeutic groups (Supplementary Fig. 7g). To be noticed, solely targeting EREG glycosylation via NGI-1 on HN4 celles in immune-deficiency mice did not show significant efficacy on both tumor weight and volume, suggesting that NGI-1 mainly affects tumor growth by affecting immune function, rather than affecting the self growth ability of tumor cells (Supplementary Fig. 7h–j). Taken together, these results indicated that combination of targeting EREG glycosylation via NGI-1 significantly enhances the efficacy of anti-PDLl blockade in vivo.

## Discussion

**Fig. 8:**
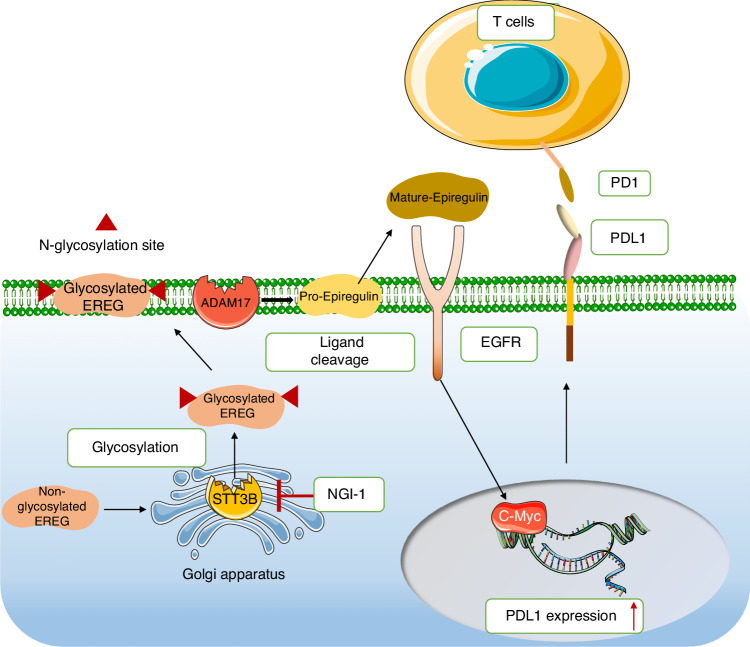
The proposed model of STT3B-mediated EREG glycosylation in promotion of immunoevasion via PDL1 upregulation in HNSCC

Epiregulin (EREG) is an EGFR ligand and is expressed at low levels in normal tissues. Elevated EREG mainly activates EGFR signaling pathways and promotes tumor progression in HNSCC cells. In this study, we showed that STT3B-mediated EREG glycosylation upregulated PDL1 in HNSCC (Fig. 8). Furthermore, combining PDL1 mAb with an STT3B-specific inhibitor enhances the efficacy of therapy of HNSCC by enhancing CD8+ T cell activity.

Our study provides several new insights into the mechanism of immunosuppression in HNSCC. First, we identified N-linked glycosylation as a key posttranslational regulation mechanism for EREG stabilization. We showed that glycosylation of EREG inhibits proteasome-mediated protein degradation. While exploring the asparagine residue involved in such regulation, we discovered that N47 is responsible for EREG glycosylation. In-depth analysis revealed that STT3B interacts with unglycosylated EREG, which accounts for aberrant EREG glycosylation in HNSCC. We showed that STT3B knockout attenuates core glycosylated EREG expression and strengthens immune response activation. Importantly, the improved efficacy of combination targeting EREG and glycosylation inhibition provide a potential therapeutic approach for translation. NGI-1 has also been reported to block EGFR and B7H4 in other cancer types.^26–28^ Notably, accumulating evidences show that cell membrane immune checkpoint proteins, such as PDL1, are glycosylated with heavy N-linked glycan moieties in human cancers.^29^ N-linked glycosylation of PDL1 maintains its protein stability and interaction with its cognate receptor, programmed cell death protein 1 (PD-1), and this in turn promotes the evasion of T-cell immunity.^30^ Previously, targeting PDL1 glycosylation was found to be effective with combination of cancer immunotherapies,^31^ which suggests that targeting glycosylation may improve the efficacy of anti-immunoevasion in tumors.

Second, our study revealed a novel molecular mechanism underlying PDL1 dysregulation in HNSCC. Because the regulation of PDL1 is crucial for immune evasion, the mechanisms underlying PDL1 dysregulation have been intensively investigated.^6^ PDL1 levels can be regulated both at the transcriptional level and posttranslational levels. For example, PDL1 can be regulated by several transcription factors, including STAT3, c-MYC, c-JUN, HIF-1a, and NF-κB.^32–34^ Moreover, PDL1 can also be regulated by posttranslational modification, including those via CSN5, GSK3β, CDK4/CDK6, and CMTM4/6, or palmitoylated via B3GNT3.^35–37^ In this study, we showed that upregulated EREG induces PDL1 via the EGFR-c-Myc axis. In support, EGF was shown to strongly induces PDL1 expression in breast cancer cells BT549 and MB-468 cells at the posttranslational level.^30^ In this study, we found that EREG induces PDL1 expression at both the transcriptional and posttranslational levels. This suggest that different mechanisms are involved in different cancer types. Of note, although EREG and EGF are both EGFR ligands, their mechanisms in activating EGFR signaling are different.^38^ In HNSCC, EREG promotes tumor progression by inducing c-Myc expression, and Myc regulates PDL1 expression at the transcriptional level.^24^ Thus, the finding of this study, stabilized EREG via N-glycosylation, provides novel mechanism of PDL1 upregulation for immunoevasion in HNSCC.

Finally, our study provides a new synergetic therapeutic strategy for treatment of HNSCC patients. Immune checkpoint blockade via an anti-PD-1 or anti-PDL1 mAb has produced substantial benefits in several advanced cancers. However, the overall response rates of anti-PD-1 or anti-PDL1 mAb therapy were reported to be less than 40%.^39^ In this study, we identified EREG as a key regulator of PDL1 expression in HNSCC. Thus, EREG can serve as a potential target for combination therapy of HNSCC. However, there have been no specific drug or small molecule inhibitors in targeting EREG yet. In this study, we show that NGI-1, which targeting STT3B-mediated glycosylation, may provide alternative strategy to target EREG. Moreover, the combination of NGI-1 and anti-PD-L1 treatment in the MTCQ1 syngeneic mouse model showed synergistic efficacy of therapy achieved compared to either treatment alone. In conclusion, we elucidated a molecular mechanism that regulates the tumor PDL1 level, and we discovered a combination therapeutic strategy for the treatment of EREG driven HNSCC.

## Conclusion

STT3B-mediated N-glycosylation is essential for stabilization of EREG, combination of NGI-1 treatment with anti-PDLl therapy synergistically enhanced the efficacy of immunotherapy of HNSCC.

## Materials and methods

### Data mining

We mined data deeply from the open-source cBioPortal (http://www.cbioportal.org) and TISIDB (http://cis.hku.hk/TISIDB) database. The data from cBioPortal was retrieved from the TCGA database, which contains DNA copy numbers, DNA methylation, mRNA and microRNA expression levels, and nonsynonymous mutations data of a variety of cancer genomic data. We used cBioPortal database to analyze genetic alterations of EREG, EGFR and PDL1 (copy number variations and single nucleotide variations) and correlations between EREG and STT3B, RPN1, GNPAT and STT3A gene expression. We used TISIDB database, which integrated multi-omics data of immune-related genes retrieved from several public data to investigate correlations between EREG expression and lymphocytes and immunomodulators.

### Mass spectrum

Materials were prepared as follows: acetonitrile(ACN, 75-05-8, Sigma), urea(CH4N2O, 57-13-6, Sigma), Tris(2-carboxyethyl) phosphine(TCEP, 75259, Sigma), iodoacetamide(IAA, 144-48-9, Sigma), ammonium bicarbonate(NH4HCO3, 1066-33-7, Sigma), trypsin(T6567, Sigma), Ultrapure water was produced on site by Millipore Simplicity System(Billerica, MA, USA). The protein was reduced in TCEP and reacted in IAA after cooling. After that, trypsin was added to the protein solution, and the digestion was performed overnight on a shaker. The digested peptides were desalted and gradient eluted with C18 (Phenomenex, 15 μm, 300 Å), and concentrated to dryness in vacuo. For further analysis, the products were loaded on Orbitrap Exploris 480 with Dionex Ultimate 3000 RSLCnano(Thermo Fisher Scientific), and the gradient value was set as follows: Loading pump mobile phase flow rate (loading): 5 μL/min; Nano pump mobile phase flow rate (separation): 300 nL/min; the proportion of Buffer B was 2% for sample loading within 12 min; the proportion of Buffer B increased linearly from 2% to 40% for elution within 188 min; the ratio of Buffer increased to 95% within 10 min, and keep it at 95% for 5 min for impurity removal; reduce the ratio of Buffer B to 2% within 5 min, and keep it to 2% until the end of the gradient. For data analysis, we used GPSeeker to build an N-glycosylation analysis database, and perform complete N-glycopeptide matching for mass spectrometry data and identification. The number of matching polypeptides (p-MPs) ≥ 5, and the peptide sequence (p-Seq.), post-translational modification (p-PTMs), and monosaccharide linkage were screened to obtain the identification list, including N-glycopeptide identification number (Identification, IDs), polypeptide sequence, N-glycosylation site, N-glycan composition and connection mode, corresponding to complete N-glycoprotein (Accession Number) and glycan structure diagnostic fragment ions and other qualitative results.

### Cell cultures

As described in our previous study,^12^ the HNSCC-derived cancer cell lines HN4, HN6, HN12, HN13and HN30 were obtained from the University of Maryland, School of Dentistry. The HEK293, CAL27 and FaDu cell lines were purchased from American Type Culture Collection (ATCC). Human oral keratinocytes (HOKs) were purchased from Chinese Beijing North Biotech. Cells were cultured in Dulbecco’s modified Eagle’s medium (DMEM; Gibco, NY, USA) supplemented with 10% fetal bovine serum (FBS), 1% glutamine, and 1% penicillin-streptomycin. FaDu cells were cultured in RPMI-1640 (Gibco, NY, USA) with 10% FBS. All cells were maintained in a humidified atmosphere with 5% CO_2_ at 37 °C.

### Plasmids transfection

As previously, EREG shRNA expression plasmids were purchased from Shanghai Era Biotech^12^ (Shanghai, China). We amplified human EREG plasmid in a HeLa cDNA library and subcloned into the vector pcDNA3.0. We generated EREG NQ mutants (N47Q, N57Q, N73Q, N90Q, N146Q, N47Q + N90Q, N47Q + N57Q + N90Q, N47Q + NN57Q + N73Q + N90Q, N47Q + N57Q + N90Q + N146Q, N47Q + N57Q + N73Q + N90Q + N146Q) using site-directed mutagenesis, which were verified by DNA sequencing. For transfection, lipofectamine 2000 (Thermo Fisher, Cat# 11668019) and plasmid with a proportion of 2.5:1 was diluted with 200 μl serum-free Opti-MEM (Thermo Fisher, Cat# 11058021) respectively. Following gentle mixing, they were incubated for 20 min and then added into cells at room temperature. After being transfected, the cells were incubated at 37 °C, in 5% CO_2_, for 8–12 h and the culture medium was then replaced with DMEM added with 10% FBS. We collected cells after 48 h of transfection.

### Reagents

Recombinant human epiregulin, PNGase F, Endo H, EGF, AREG, and TGF-α were purchased from R&D Systems (MN, USA). Tunicamycin (TM), carboxylic acid (JQ-1), Bix, AG490 were purchased from MCE (USA). Bortezomib, Benzyl, Chloroquine(CQ), Cycloheximide (CHX), Carfilzomib, MG132, GSI, NGI-1, Gefitinib, AG1478 were purchased from Selleck (USA). They were added in cells according to manufacturer’s protocols.

### Transfection of siRNAs

Cells were transfected with 100 nmol/L siRNAs, which were used to knock down EREG expression. siRNAs were chemically synthesized by Shanghai GenePharma Co. (Shanghai, China). According to the manufacturer’s instructions, siRNAs were diluted in Opti-Eagle’s minimal essential medium (Invitrogen, CA, USA) using Lipofectamine 2000 (Invitrogen, CA, USA) as we did previously,^12^ with nontargeted siRNA being a negative control. For further analysis, the cells were exposed to different treatments after transfection and lysed with NP-40 lysis buffer. The sequences of siRNAs against the EREG sequence were as follows: siRNA-EREG (#1)-F: 5′-GCUCAAGUGUCAAUAACAAdTdT-3′ and R: 5′-UUGUUAUUGACACUUGAGCdTdT-3′; (#2)-F: 5′-CCACCAACCUUUAAGCAAAdTdT-3′ and R: 5′-UUUGCUUAAAGGUUGGUGGdTdT-3′; and (#3)-F: 5′-CUUUGACCGUGAUUCUUAUdTdT-3′ and R: 5′-AUAAGAAUCACGGUCAAAGdTdT-3′. The siRNAs against the EGFR sequence were as follows: siRNA-EGFR(#1)-F: 5′-GUCGCUAUCAA GGAAUUAAdTdT-3′ and R: 5′-UUAAUUCCUUGA UAGCGACdTdT-3′; (#2)-F: 5′-GGCUUGCAUUGA UAGAAAUdTdT-3′ and R: 5′-AUUUCUAUCAAU GCAAGCCdTdT-3′; and (#3)-F: 5′-GUCCGCAAG UGUAAGAAGUdTdT-3′ and R: 5′-ACUUCUUACA CUUGCGGACdTdT-3′.

### Flow cytometry

Fresh tumors were mechanically dissociated after being obtained. Briefly, after mechanical disaggregation, we used RPMI 1640 (Sigma-Aldrich), which includes DNase I (20 U/mL; Sigma-Aldrich), Collagenase I (1 mg/mL; EMD Millipore) and Collagenase IV (250 U/mL; Worthington Biochemical Corporation) for tumors digestion to make single cell suspensions. The binding of antibodies to FcγIII/II receptor was blocked by using anti-mouse CD16/CD32 monoclonal antibodies (mAbs; 2.4G2, BD Biosciences, USA) in isolated lymphocytes, which were then stained with a fixable viability dye (Thermo Fisher Scientific, USA) to exclude dead cells. Fluorochrome-conjugated antibodies were then used to stain cells for 15 min at room temperature and incubated with a fixation/permeabilization solution (Thermo Fisher Scientific, USA). Brefeldin A (GolgiPlug, BD Biosciences, USA) and monensin (GolgiSTOP, BD Biosciences) were used to stimulate cells according to the manufacturer’s protocol. The antibodies were obtained from BD Biosciences (PD-1, J43; IFN-γ, XMG1.2) and BioLegend (CD8, 53-6.7). All flow cytometric analyses were performed using an LSR II system (BD Biosciences, USA) and FlowJo software (Tree Star Inc.).

### Elisa

Elisa kit was obtained from R&D systems (USA). Supernate samples of transfected cells were collected for Elisa analysis. According to the manufacture’s protocol, capture antibody diluted in PBS was incubated in plates with 100 μL each well for overnight at a room temperature and then washed with washing buffer for 3 times. After being blocked for 1 h and washed for 3 times, plates were added in samples and standards and incubate for 2 h and then washed for 3 times at room temperature. Detection antibody was added for incubation for 2 h. Streptavidin-HRP and substrate solution was added and incubated for 20 min sequentially. Stop solution were added then. After all procedures finished, a microplate reader set to 450 nm was used to determine the optical density of each well immediately.

### Immunohistochemistry (IHC) staining

Formalin-fixed, paraffin-embedded (FFPE) HNSCC specimens was obtained from the Ninth People’s Hospital (Shanghai, China). And then, HNSCC micro-tissue array, which contains 124 case was made. Immunohistochemistry (IHC) staining was performed on a 4-μm tumor section cut from the micro-array for EREG (R&D Systems, MN, USA) CD8 (Novocastra, Newcastle, UK), STT3B (Proteintech. Co, Ltd), PDL1(clone 22c3, DAKO, Santa Clara, CA) staining. Tissue sections were deparaffinized and received antigen retrieval using sodium citrate for 30 min. After being blocked using hydrogen peroxide for 10 min, the tissue samples were incubated with primary antibody overnight at 4 °C after washed. In the second day, slides were incubated using biotinylated-conjugated antibody for 30 min. Washed again, slides were then stained using 3,3′-diaminobenzidine tetrahydrochloride solution and counterstained with Mayer’s hematoxylin for 2 min. H-score is calculated using the formula: H-score = 3x% of strongly stained cells + 2 y% of intermediately stained cells + 1z% of weakly stained cells while x, y, z represents for appropriation of each cell, ranging from 0 to 300. Cells staining was evaluated by Case-viewer software (3DHISTECH.Ltd) for H-score calculation. The score was evaluated by two pathologists independently.

### Immunofluorescence (IF)

As we did previously,^12^ After being rinsed with PBS three times, cancer cell lines were fixed with 3.7% formaldehyde, after which they were permeabilized with 0.1% Triton X-100. After cells being blocked with 1% BSA for 1 h, the primary antibody was used for cells’ incubation for overnight. The cells were then incubated with Alexa Fluor 488 or 594 donkey anti-rabbit IgG antibody (Invitrogen, NY, USA) in the dark for 1 h at room temperature. After being washed with PBS (containing 0.02% Tween 20) for 3 times, the cells were stained by using aqueous mounting medium which contains 0.5 mg/mL 4′,6′-diamidino-2-phenylindole and evaluated by using a fluorescence microscope.

### Western blotting (WB) and immunoprecipitation (IP)

Samples were lysed by NP-40 lysis buffer purchased from Beyotime Biotech (Jiangsu, China) on ice. After sonication, all cell lysates received centrifugation at 12 000 r/min for at least 20 min at 4 °C and bicinchoninic acid (BCA) detection for protein concentration. After being denatured at 100 °C for 5 min with loading buffer (Beyotime Biotech, Jiangsu, China), samples were separated using 10% sodium dodecyl sulfate-polyacrylamide gel by electrophoresis (30 min for 120 volts followed by 60 min for 60 volts) and then transferred to a nitrocellulose membrane (90 min for 100 volt) for further WB. The membrane was then blocked using 5% skim milk, prepared with ddH_2_O, for 1 h. Primary antibody was then used for incubation at 4 °C for overnight. All primary antibodies were illustrated at IHC and IF section. 5 μg of antibodies against HA, Flag, EREG, STT3B or negative control IgG were used for IP with 1.0 mg of whole-cell lysates, which were then used for further immunoblotting. Anti-GAPDH antibody was obtained from Santa Cruz Biotechnology (CA, USA). Antibodies against EREG, c-Myc, EGFR, Flag, p-EGFR and HA were obtained from Cell Signaling Technology (MA, USA). Antibodies against STT3B was purchased from Proteintech Group (USA). On the second day of primary antibody incubation, the membrane was incubated by using HRP-labeled goat anti-rabbit antibody or murine IgG (CST, 1: 500) for 2 h at room temperature. 1X TBST was used for washing after each procedure. Western chemiluminescence HRP substrate (Beyotime Biotech, Jiangsu, China) was used for specific protein expression observation. Every experiment was repeated for 3 times.

### Quantitative polymerase chain reaction (q-PCR)

Total RNA samples were extracted with TRIzol Reagent (Invitrogen, CA, USA) using ultraviolet spectrophotometer to detect RNA concentration, and cDNA was prepared from using a PrimeScriptTM RT Reagent kit (TaKaRa, Kyoto, Japan) as previously.^40^ mRNA levels were measured by q-PCR with the following primers: EREG (F: 5′-ATCCTGGCATGTGCTAGGGT-3′ and R: 5′-GTGC TCCAGAGGTCAGCCAT-3′); and GAPDH (F: 5′-TCCACCACCCTG TTGCTGTA-3′ and R: 5′-ACCACAGTCCATGCCA TCAC-3′), and qRT–PCR was conducted using qPCR SYBR Green Master Mix (Yeasen, Shanghai, China) following the manufacturer’s instructions.

### Predication model of structure of EREG-WT and EREG-N47Q

We submitted the sequence to the robetta server, construct the three-dimensional model of the protein using robettafold. We obtained the models of the five proteins, then submit the first model to the saves6.0 database, then visualize the three-dimensional structure of the protein using PyMOL. We found the polar uncharged asparagine and its surrounding polar interactions at position 47 using PyMOL, and then use the MUTAGENESI module to carry out site mutation. After that, we used the sculling module to optimize the concept after mutation, and finally analyzed the changes of interaction.

### In vivo therapeutic assay

Approved by the Animal Ethics Committee of Ninth People’s Hospital, animal studies were performed in accordance with guidelines from the Shanghai Jiao Tong University School of Medicine. NGI-1 was diluted with 10% DMSO + 40% PEG300 + 5% Tween 80 + 45% saline solution with a final concentration of 1 mg/mL, while PDL1 inhibitor (#A2115,Selleck, USA) was diluted in PBS at 0.5 mg/mL. In the animal study, female SPF C57BL/6 mice (6 weeks old) were obtained from the Shanghai Laboratory Animal Center (Shanghai, China). MTCQ1cells were used for tumor xenograft model establishment, and the cells (5 × 10^6^ cells per 100 μL of PBS) were subcutaneously injected into the flanks of mice, and the tumor sizes were monitored three times a week. The tumor sizes were measured according to the formula: (A)(B^2^)π/6, where A indicates the length of the tumor, and B is the width of the tumor. NGI-1 and PDL1 inhibitor were administered via abdominal injection 4 times a week at a dosage of 10 mg/kg and 5 mg/kg respectively. All mice were euthanized at the end of the study. Differences in the tumor volume and tumor weight among were analyzed by using Chi-square test for all two groups. Organ tissues of each mice including heart, spleen, liver, lung were collected, formalin embedded, and stained by Hematoxylin and eosin (H&E) to evaluate medicine toxicity. PDL1, CD8 and Ki-67 IHC staining and evaluation of each tumor were performed according to the procedure mentioned above.

### T cell mediated tumor cell killing assay

Spleen of C57BL/6 mice was sufficiently grinded to make single cell suspensions by passing the suspensions through 70 μm nylon mesh, and CD8 T cells were obtained using CD8^+^ T cell isolation kit (Order number 130-104-075, Miltenyi Biotec, Germany) and then activated with CD3/CD28 antibody dynabeads (#11456D, Themo Fisher Scientific, USA) for 3 days. SCC7 and MTCQ1 cells were co-cultured with T cells with a ratio of 1:5 for 3 days. To assess T cell mediated cell killing ability on the condition of NGI-1 stimulation, T cells were washed using PBS, and surviving tumor cells were fixed and stained with crystal violet solution.

### TdT-UTP nick end labeling (TUNEL)

TUNEL kit was obtained from Beyotime. Co, Ltd and TUNEL staining was performed kit according to the manufacturer’s instructions. Briefly, the deparaffined tumor sections were incubated by proteinase K for 20 min and then washed by PBS. TUNEL detection liquid was used for incubation for 1 h at 37 °C. A fluorescent microscope was used to image TUNEL-positive cells, which was imaged with green fluorescence and defined as apoptotic cells.

### Statistical analysis

The data were analyzed using SPSS statistical software program (SPSS, Statistics 26, IBM). Each Experiment was repeated at least twice and results were expressed as mean ± SEM unless otherwise specified. The significance of the differences in the assays was tested by Student *t*-test or one- or two-way ANOVA. For non-parametric data, statistical significance was tested by Mann-Whitney test or Kruskal-Wallis test followed by selected comparison by Dunn’s multiple comparison tests with multiple comparison correction. All statistical tests were two-sided, and a P-value of 0.05 or less was considered to be statistically significant.

### Supplementary information

Supply Figures and language edited certificate

## Data Availability

All datasets on which the conclusions of the paper rely are available to readers.
