# Supplementary material for: Stabilization of EREG via STT3B-mediated N-glycosylation is critical for PDL1 upregulation and immune evasion in head and neck squamous cell carcinoma
Source: Int J Oral Sci. 2024 Jul 1;16:47. doi: 10.1038/s41368-024-00311-1 (PMC11214941; doi:10.1038/s41368-024-00311-1)
Supplement: Supplementary file 1 — Supply Figures and language edited certificate [file 41368_2024_311_MOESM1_ESM.pdf]

Supplementary information

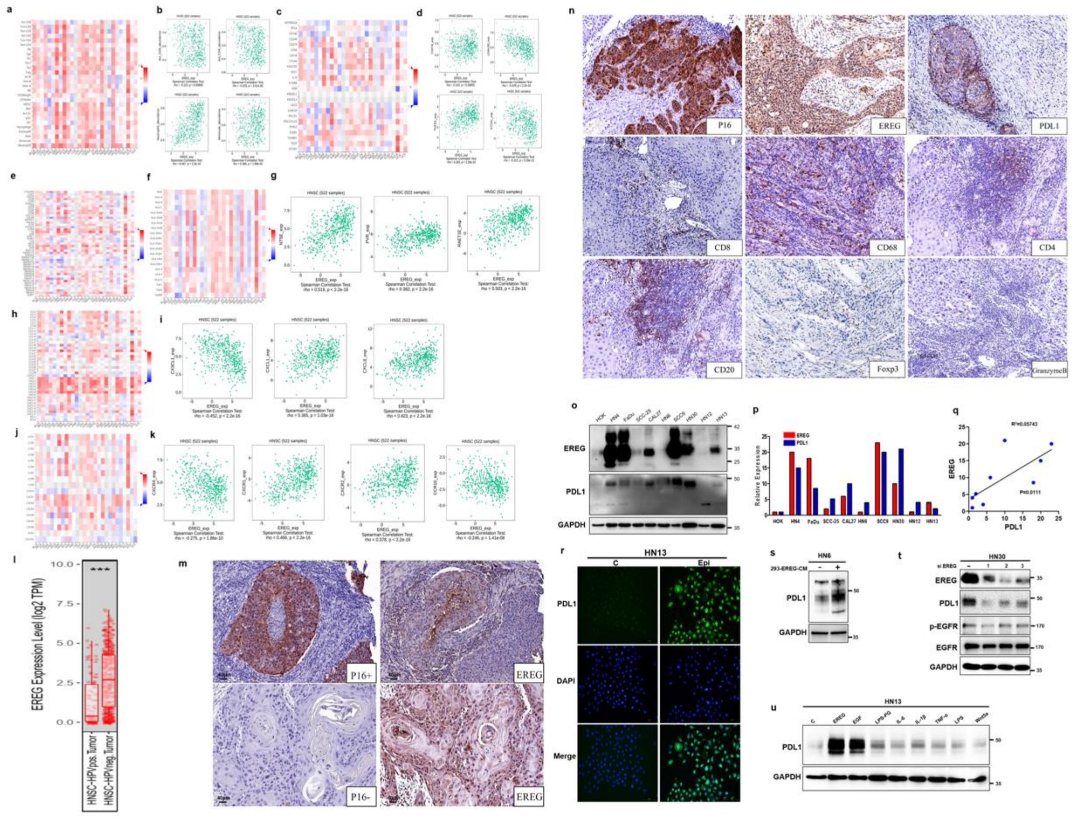

Supplementary Fig. 1. Related to Figure 1.

(a) The landscape of the relationship between EREG expression and TILs in different types of cancer. Red indicates positive correlation, and blue indicates negative correlation. **(b)** Top 4 TILs displaying the greatest Spearman's correlation with EREG expression. **(c)** The landscape of the relationship between EREG expression and the abundance of immuno-inhibitors in different types of cancer. **(d)** Top 4 immunoinhibitors displaying the greatest Spearman's correlation with EREG expression. **(e-k)** The landscape of the relationship between EREG expression and immuno-stimulators, MHCs, chemokines, and receptors in different types of cancer. Red indicates positive correlation, and blue indicates negative correlation. Top 3

immuno-stimulators, chemokines, and receptors displaying the greatest Spearman's correlation with EREG expression. **(l)** Analysis of the expression level of EREG in HPV (-) HNSCC and HPV (+) HNSCC by using TIMER. **(m)** Representative images of P16 and EREG staining in two patients with different HPV status. (**n**) Representative images of P16, EREG, PDL1, CD8, CD68, CD4, CD20, Foxp3 and Granzyme B staining. **(o-q)** EREG expression was positively correlated with PDL1 (R^2= 0.05743, p=0.0111) expression in HNSCC cell lines. **(r)** Immunofluorescence staining for PDL1 in HN13 cells treated with or without 50 ng/mL epiregulin. **(s)** Western blot analysis of PDL1 from HN6 cells treated with conditional medium from HEK293-EREG-WT cells for 24 h. **(t)** Western blot analysis of EREG, PDL1 and EGFR expression in HN30 cells after transfection with siEREG or siNC siRNAs. **(u)** Western blot analysis of PDL1 expression in HN13 cells treated with different cytokines.

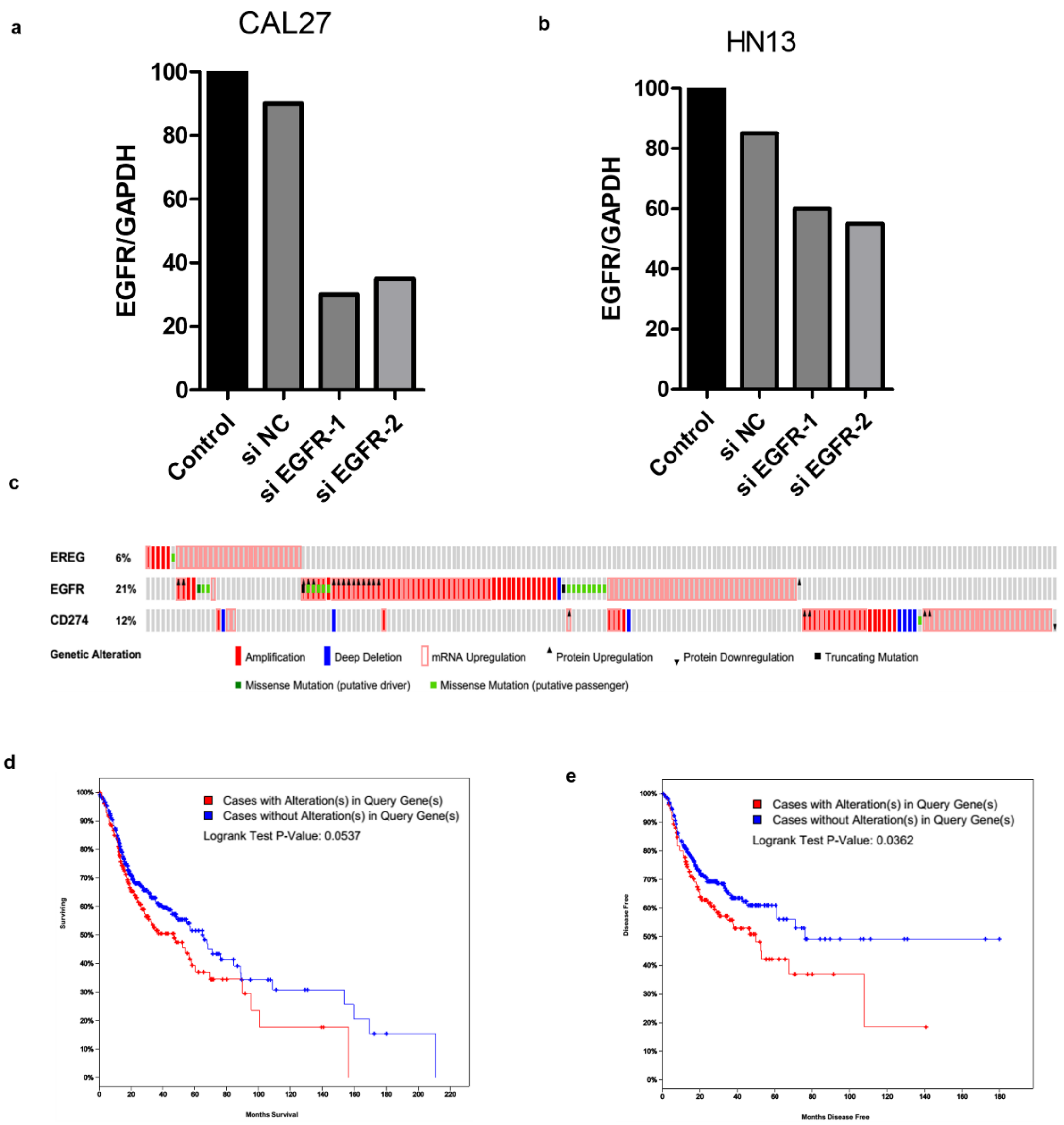

Supplementary Fig. 2. Related to Figure 2.

(a-b) Ratio of EGFR and GAPDH in CAL27 and HN13 cells. **(c)** OncoPrint of EREG-EGFR-PDL1 pathway alterations in HNSCC. Genomic alterations of different members of this pathway were mutually exclusive (upper panel). **(d-e)** Patient survival data obtained from TCGA were analyzed based on the mRNA expression level of EREG-EGFR-PDL1.

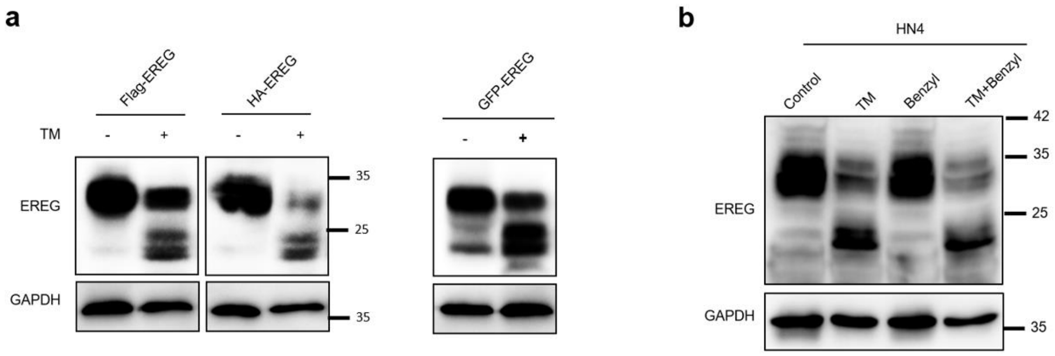

Supplementary Fig. 3. Related to Figure 3.

(a) Immunoblot of EREG in HEK293-EREG cells treated with TM (N-linked glycosylation inhibitor) as indicated. **(b)** Immunoblot of EREG in HN4 cells treated with TM or benzyl (O-linked glycosylation inhibitor) as indicated.

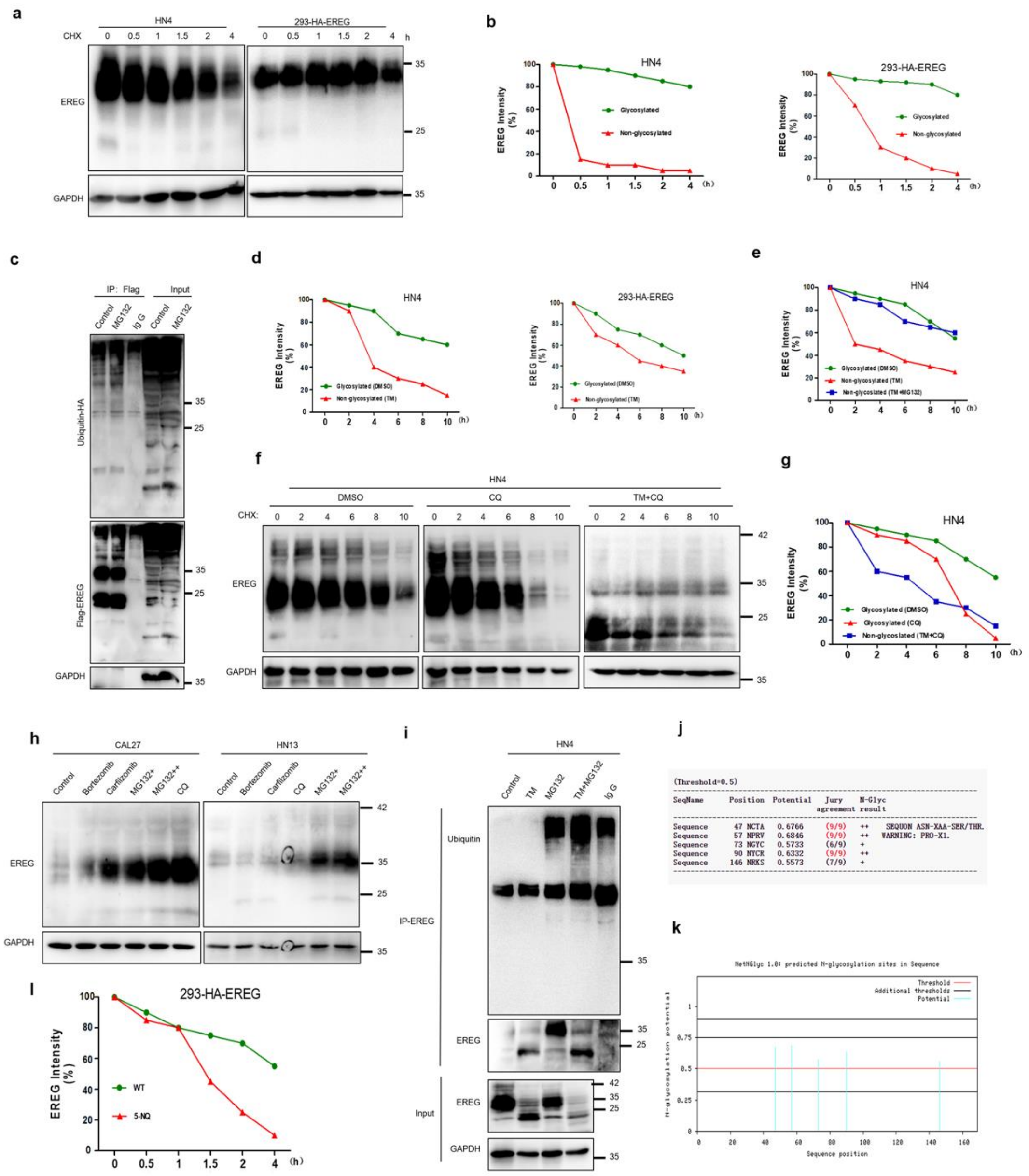

Supplementary Fig. 4. Related to Figure 4.

(a) Pulse-chase analysis of HN4 and 293-EREG cells. Cells were treated with 100 μg/mL cycloheximide (CHX) at the indicated time points. EREG levels were measured by immunoblotting. GAPDH was used as a loading control. **(b)** Graphic representation of the densitometry results of EREG after cycloheximide (CHX) treatment in Supplementary Figure 4a. Circle indicates glycosylated EREG with DMSO, and square indicates nonglycosylated EREG with TM. **(c)** Ubiquitination assay. FLAG-EREG and

HA-Ubiquitination were together transfected into 293T cells in the presence or absence of the MG132 proteasome inhibitor. FLAG-EREG was then immunoprecipitated by anti-FLAG followed by immunoblotting using an antibody against ubiquitin. **(d)** The intensity of the glycosylated form versus the nonglycosylated form of EREG in Figure 4a and 4b was quantified using ImageJ software. **(e)** The intensity of the glycosylated form versus the nonglycosylated form of EREG in Figure 4c was quantified using ImageJ software. **(f)** Immunoblot of EREG in HN4 cells treated with CHX for the indicated time in the presence or absence of TM and CQ. **(g)** The intensity of the glycosylated form versus the nonglycosylated form of EREG was quantified using ImageJ software. **(h)** Immunoblot of EREG in CAL27 and HN13 cells treated with different inhibitors as indicated. **(i)** Ubiquitination of EREG proteins in HN4 cells treated with TM or MG132 as indicated. EREG proteins were immunoprecipitated with EREG antibody and then immunoblotted with ubiquitin antibody. **(j)** Schematic diagram showing the mutation constructs of EREG. **(k)** Predicted N-glycosylation sites of human EREG by NetNGlyc1.0 Server. **(l)** The intensity of the glycosylated form versus the nonglycosylated form of EREG in Figure 4i was quantified using ImageJ software.

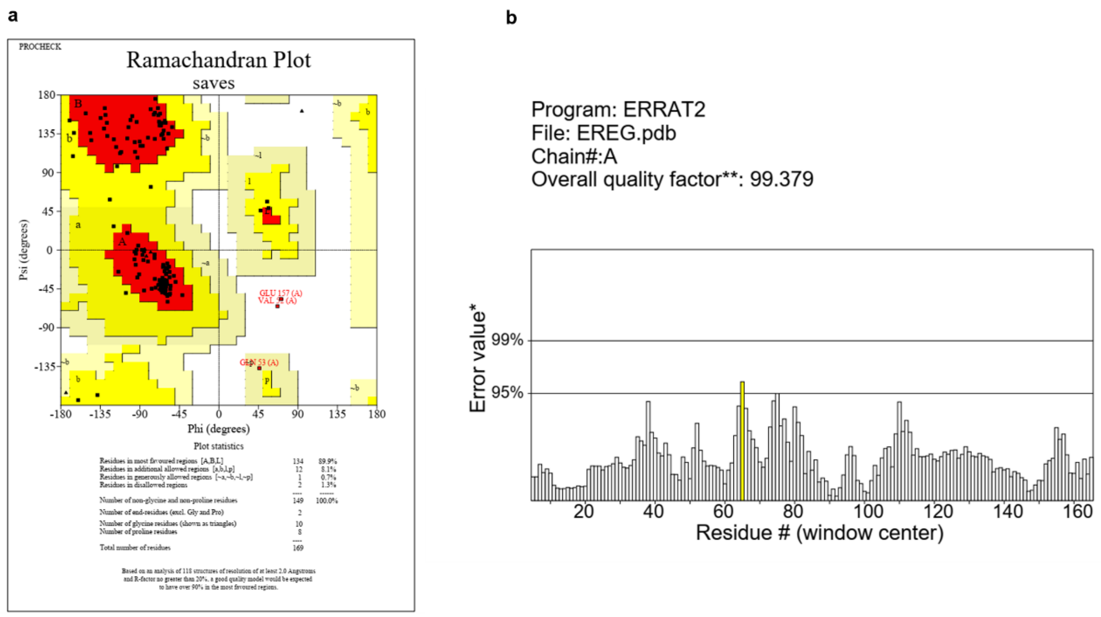

Supplementary Fig. 5. Related to Figure 5.

(a) Model evaluation: the calculated Raman spectrum is shown. From the Laplace conformation, we can see that 98.7% of the amino acid residues are in the reasonable region (including 89.6% in the optimal region, 8.7% in the acceptable region, and 0.7% in the general allowable region). The amino acid residues in the non allowable region account for 1.3%. Therefore, based on the evaluation principle that the reasonable region and the allowable region are greater than 90%, the model is reasonable. **(b)** 99.379% of the protein model meets the errat verification through errat scoring, which meets the requirements of the program with an evaluation greater than 91. According to Ramachandran plot and errat scores, the constructed protein structure has high reliability and can be used as a template for subsequent studies.

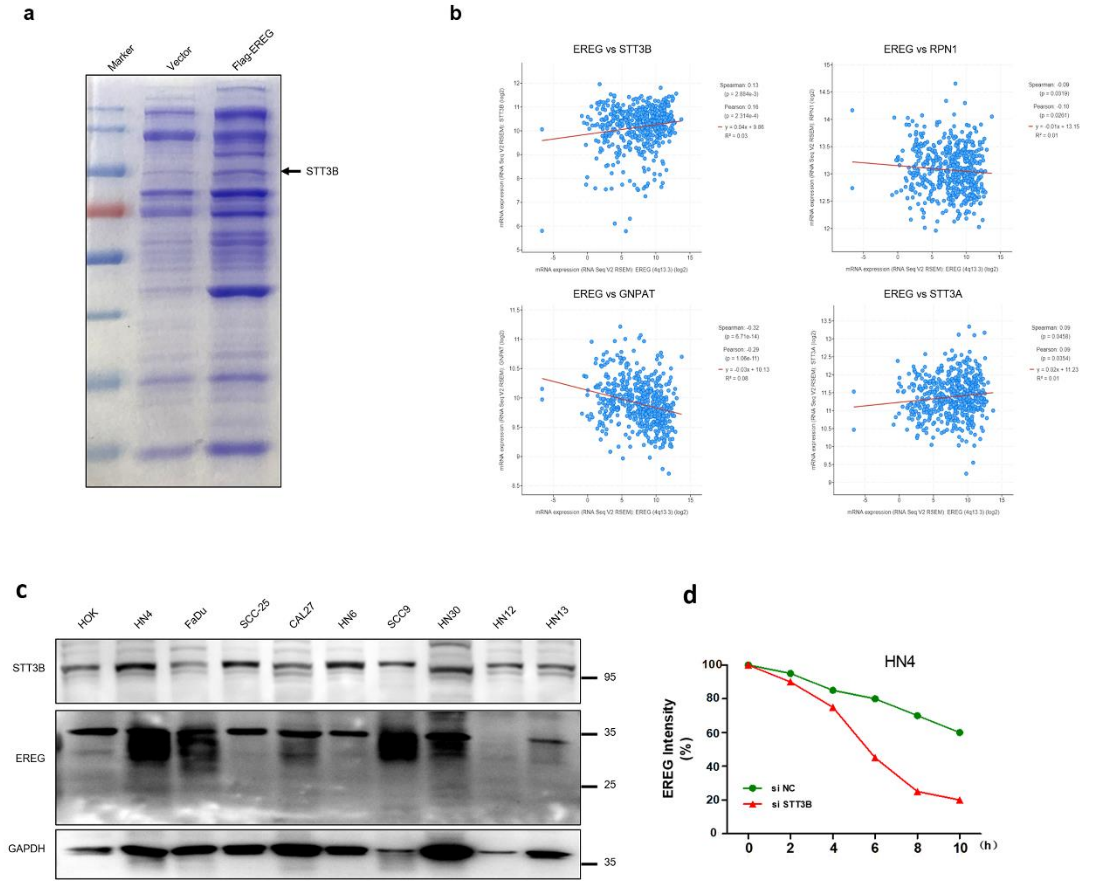

Supplementary Fig. 6. Related to Figure 6.

(a) Flag-EREG or vector control was transfected into HEK293 cells. The EREG complex was then purified followed by mass spectrometry analysis. Coomassie blue staining of the purified EREG immunocomplex is shown. The STT3B glycosyltransferase was identified. **(b)** Pearson correlation coefficients between EREG and several glycosyltransferases across HNSCC samples from TCGA database. **(c)** The protein expression of STT3B and EREG in 10 HNSCC cell lines was measured by immunoblotting. **(d)** The intensity of EREG in HN4 cells after transfection with siSTT3B or siNC siRNAs was quantified using ImageJ software.

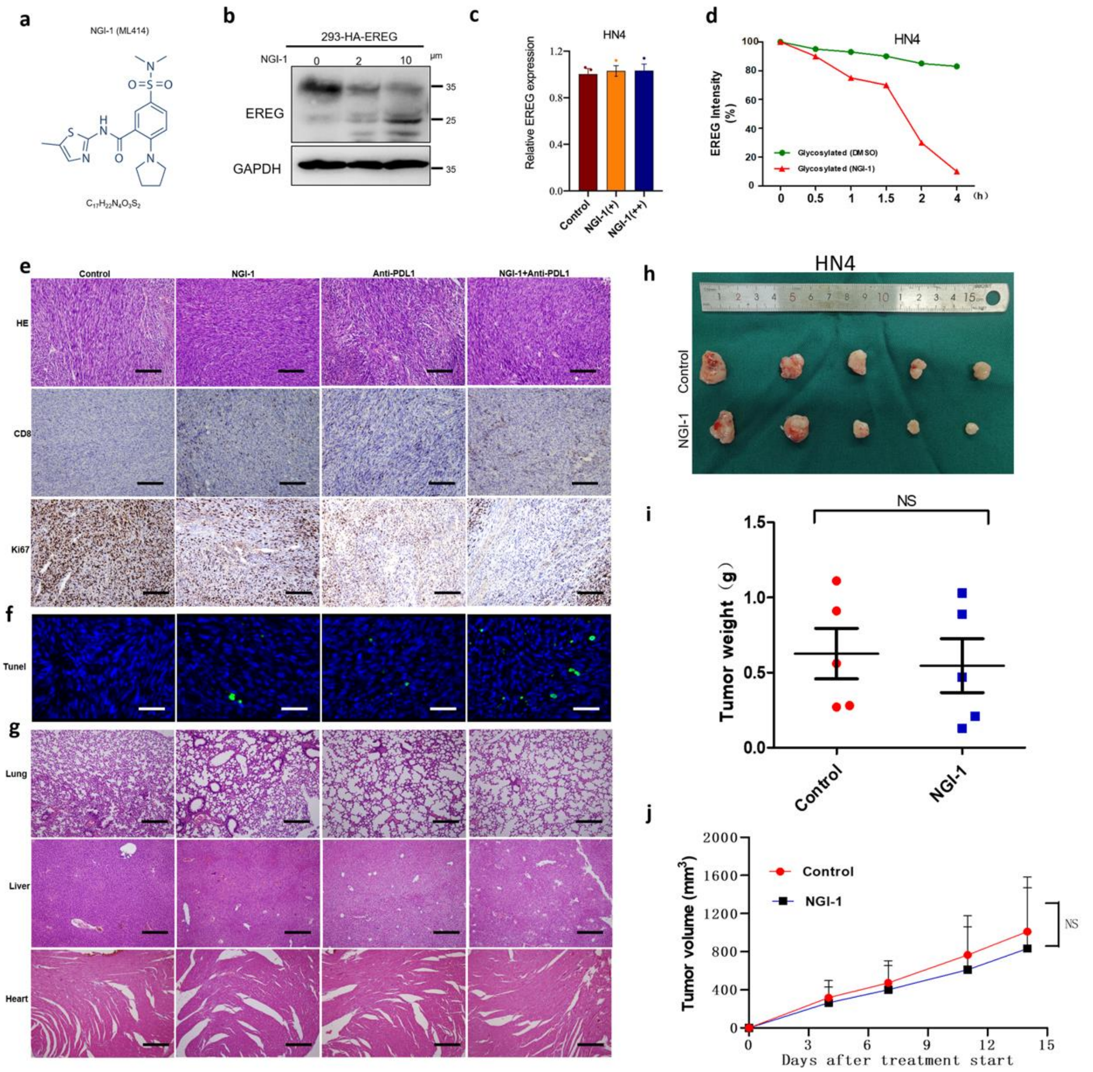

Supplementary Fig. 7. Related to Figure 7.

(a) Structure diagram of NGI-1. **(b)** HEK293-HA-EREG cells were treated with NGI-1 for 24 hours, and the expression of EREG was examined by immunoblotting. **(c)** HN4 cells were treated with NGI-1 for 24 hours, and the mRNA expression of EREG was examined by Q–PCR. **(d)** The intensity of the glycosylated form versus the nonglycosylated form of EREG was quantified using ImageJ software. **(e)** Representative immunohistochemical staining for HE, Ki-67 and CD8 in MTCQ1 xenograft tumors treated with vehicle, NGI-1, anti-PDL1 mAb, or NGI-1 + anti-PDL1 mAb. **(f)** Representative photographs of TUNEL staining in different groups. **(g)**

Representative images showing H&E staining of mouse lungs, livers, and hearts from the indicated groups. **(h)** HN4 cells were injected into nude mice. When established tumors were palpable, the mice were treated with vehicle or NGI-1. MTCQ1 tumors in each group were harvested and photographed at the end of the experiment. Photographs of the xenograft tumors are shown. **(i)** Tumor weights were measured for each treatment group at autopsy. **(j)** Tumors were measured with calipers, and values were plotted. The vertical bars indicate the mean tumor size (mm^3) ± SE.

AJE®

Editing Certificate

This document certifies that the manuscript

Stabilization of EREG via STT3B-mediated N-glycosylation is critical for PDL1 upregulation and immune evasion in head and neck squamous cell carcinoma

prepared by the authors

Shengming Xu, Haifeng Wang, Yu Zhu，Yong Han, Liu Liu, Xiangkai Zhang，Jingzhou Hu，Wuchang Zhang, Shengzhong Duan，Jiong Deng，Zhiyuan Zhang, Shuli Liu

was edited for proper English language, grammar, punctuation, spelling, and overall style by one or more of the highly qualified native English speaking editors at AJE.

This certificate was issued on **February 2, 2022** and may be verified on the AJE website using the verification code **6EE7-89C6-25BC-64B2-F566**.

Neither the research content nor the authors' intentions were altered in any way during the editing process. Documents receiving this certification should be English-ready for publication; however, the author has the ability to accept or reject our suggestions and changes. To verify the final AJE edited version, please visit our verification page at aje.com/certificate.
If you have any questions or concerns about this edited document, please contact AJE at support@aje.com.

AJE provides a range of editing, translation, and manuscript services for researchers and publishers around the world.
For more information about our company, services, and partner discounts, please visit aje.com.
